# Inter-kingdom interactions and stability of methanogens revealed by machine-learning guided multi-omics analysis of industrial-scale biogas plants

**DOI:** 10.1038/s41396-023-01448-3

**Published:** 2023-06-07

**Authors:** Roland Wirth, Zoltán Bagi, Prateek Shetty, Márk Szuhaj, Teur Teur Sally Cheung, Kornél L. Kovács, Gergely Maróti

**Affiliations:** 1 Institute of Plant Biology, Biological Research Centre, Szeged, Hungary; 2 Department of Biotechnology, University of Szeged, Szeged, Hungary; 3 Faculty of Water Sciences, University of Public Service, Baja, Hungary

**Keywords:** Metagenomics, Industrial microbiology, Metagenomics

## Abstract

Multi-omics analysis is a powerful tool for the detection and study of inter-kingdom interactions, such as those between bacterial and archaeal members of complex biogas-producing microbial communities. In the present study, the microbiomes of three industrial-scale biogas digesters, each fed with different substrates, were analysed using a machine-learning guided genome-centric metagenomics framework complemented with metatranscriptome data. This data permitted us to elucidate the relationship between abundant core methanogenic communities and their syntrophic bacterial partners. In total, we detected 297 high-quality, non-redundant metagenome-assembled genomes (nrMAGs). Moreover, the assembled 16 S rRNA gene profiles of these nrMAGs showed that the phylum *Firmicutes* possessed the highest copy number, while the representatives of the archaeal domain had the lowest. Further investigation of the three anaerobic microbial communities showed characteristic alterations over time but remained specific to each industrial-scale biogas plant. The relative abundance of various microorganisms as revealed by metagenome data was independent from corresponding metatranscriptome activity data. *Archaea* showed considerably higher activity than was expected from their abundance. We detected 51 nrMAGs that were present in all three biogas plant microbiomes with different abundances. The core microbiome correlated with the main chemical fermentation parameters, and no individual parameter emerged as a predominant shaper of community composition. Various interspecies H_2_/electron transfer mechanisms were assigned to hydrogenotrophic methanogens in the biogas plants that ran on agricultural biomass and wastewater. Analysis of metatranscriptome data revealed that methanogenesis pathways were the most active of all main metabolic pathways.

## Introduction

In engineered anaerobic digestion systems (AD), the decomposition of organic matter and biogas production are based on efficient nutrient recycling [1, 2]. This process involves a diverse microbial community, with each microbe having a specific role. The composition and function of the microbial community involved in the stages of AD play an important role in the efficiency of the overall process, which is also influenced by numerous factors, such as microbe-microbe interactions, substrate composition, physicochemical parameters, and operating conditions [3–6].

Unravelling microbial interactions and their underlying mechanisms is an intricate task since many microbes cannot survive without specific microbial partners [7]. Innovative methods for microbial isolation and cultivation have been developed, with some proving successful in bringing novel microorganisms into culture [8]. However, further development and advancement of these technologies are needed to unlock the full potential of microbial diversity [9, 10]. Since there are strong syntrophic interactions between the microbes in AD microbial consortia, in-depth research is required to understand their diversity, metabolic role, and distribution. These studies have been initially hindered by limitations inherent to culture-dependent microbiological methods, which require the isolation of microorganisms and can be challenging when syntrophic relationships are ubiquitous [11–13]. High-throughput sequencing and bioinformatics tools permit bulk analysis of genomic material and thereby provide insight into the taxonomy and functions of entire microbial communities [14–16].

The identification and genome characterization of commonly found microbes in AD microbiomes can reveal crucial pathways and ecological characteristics involved in the microbial food chain [17, 18]. Previous studies using 16 S rRNA gene amplicon sequencing showed that core microorganisms create a stable community capable of resisting various perturbations (i.e., AD parameter changes) [19–21]. However, amplicon sequencing and read-based metagenomics approaches may be unable to identify unknown species by core microbiome analysis due to their dependence on reference databases [22, 23]. Additionally, biogas-producing communities represent a large and diverse contingent of uncharacterised microorganisms that have previously been described as a “microbial dark matter” [19, 24, 25]. To address this issue, increasingly sophisticated bioinformatic algorithms can be used to reconstruct the genomes of individual species (or MAGs: metagenome-assembled genomes) from these complex communities [26–28].

Several recent studies employing genome-resolved metagenomics have recovered characteristic MAGs from lab- and industrial-scale biogas reactors [23, 29–31]. These studies revealed that *Bacteroides* and *Firmicutes* have versatile interactions with hydrogenotrophic methanogens through their H_2_, CO_2_, and formate-producing abilities [17, 32]. Moreover, ammonia concentration is the main parameter shaping the methanogen community [29]. It is necessary to reconstruct genome fragments from the metagenome in an efficient and accurate manner to explore these interactions in silico [33, 34]. Recently published studies demonstrated that semi-supervised machine learning could significantly improve binning performance [35, 36], which – combined with metatranscriptomics approach – may enable more in-depth examination of microbial interactions in different environments.

The present study investigated the structure and function of microbiomes in three industrial-sized biogas reactors, each fed with a distinct, characteristic substrate. Each reactor was monitored at seasonal intervals over a one-year period. Shotgun sequencing followed by machine learning-guided genome-centric metagenomic and metatranscriptomic analysis framework were used to identify microbial composition and ongoing metabolic activities. The shared portion of the microbial community involved in digesting heterogeneous substrates was investigated using an occurrence-based core community concept [37]. The main objective of this research was to uncover the relationship between the abundant core methanogenic population and particular syntrophic bacteria. More specifically, we focus on key networks of interspecies syntrophy and find an intriguing correlation between chemical fermentation parameters and the core anaerobic microbiota present in the reactors.

## Materials and methods

### AD samples

Samples were taken from three state-of-the-art anaerobic digesters in Hungary. Two of the digesters are in Szeged (MWBP, and SZBP), and the other is in Kecskemét (KBP). Key characteristics of each biogas plant studied here are summarised in Suppl. Table 1. These biogas plants were selected using the following criteria: (i) the digesters have been operating without issue for more than five years; and (ii) the digesters use distinct biopolymers as the main substrate of decomposition for biogas production. Sampling was performed for a one-year period at the following (seasonal) intervals: October 2020, January 2021, April 2021, and July 2021. Samples were directly transported to the laboratory and were processed immediately on upon arrival. AD parameter measurements, and DNA/RNA purification were performed on fresh biogas plant (BP) samples in triplicate (i.e., biological triplicates: *n* = 3).

### Determination of AD chemical parameters

For each BP, we measured: sludge pH, carbon to nitrogen ratio (C/N), total solid (TS) content, volatile solid (VS) content, total ammonia nitrogen (TAN; i.e., ammonium ions and dissolved ammonia), volatile organic acid (VOA) content, and total inorganic carbon (TIC). Measurements were taken as previously published [38].

Biochemical methane potential (BMP) batch tests were performed in 160 mL reactor vessels (Wheaton glass serum bottle, Z114014 Sigma-Aldrich) containing 60 mL of liquid phase. The inoculum (BP content filtered to remove particles larger than 2 mm) to substrate (α-cellulose: C8002 Sigma-Aldrich) ratio was set according to the VDI 4630 standard (Vereins Deutscher Ingenieure 4630, 2006) at the inoculum to substrate VS ratio = 2:1. A detailed description of the gas sampling and measurement procedures can be found in a previous publication [39].

### Total DNA and RNA purification

Aliquots of 2 mL were obtained from the samples of each BP for total community DNA and RNA isolation. Purification was performed in triplicate, and the resulting extractions were pooled together. All extractions were carried out using ZymoBIOMICS DNA/RNA miniprep kits (R2002, Zymo Research, Irvine, USA). After lysis (bead homogenisation was performed using a Vortex Genie 2 with a bead size of 0.1 mm, a homogenisation time of 15 min, and at max speed), the Zymo Research kit parallel DNA and RNA purification protocol was followed. DNA and RNA quantities were estimated using an Agilent 2200 TapeStation (Agilent Technologies, Santa Clara, USA).

### Metagenome, and metatranscriptome sequencing

We closely followed all manufacturer recommendations for the Illumina sequencing platform (Illumina Inc., San Diego, USA). Pooled genomic DNA samples were used to sequence libraries constructed using the NEBNext Ultra II Library Prep Kit (NEB, Ipswich, USA). Paired-end metagenomics sequencing was performed on an NextSeq 550 (Illumina) sequencer using the NextSeq High Output Kit v2 sequencing reagent kit. Metatranscriptome sequencing from pooled RNA samples was performed as follows: libraries were first prepared using a Zymo-Seq RiboFree Total RNA Library Kit, which includes a universal rRNA depletion step. Paired-end mRNA sequencing was then performed on a NextSeq 550 (Illumina) sequencer using the NextSeq High Output Kit v2 sequencing reagent kit. Primary data analysis (i.e., base-calling) was performed using “bcl2fastq” software (version 2.17.1.14, Illumina). Characteristic fragment parameters are summarised in Supplimentary Table 2.

### Metagenome assembly and binning

Raw sequences were filtered by fastp (version 0.23.2, length required: 150 bp) and checked with FastQC (version 0.11.8). The filtered sequences produced by fastp were then co-assembled separately by Megahit (version 1.2.9, 4 samples per BP = 3 co-assemblies). The settings used were as follows: min contig length = 1500; min k-mer size = 21; max k-mer size = 141 [40]. The metagenomics binning procedure was performed separately for each AD metagenomics dataset until the dereplication step. We used Anvi’o (version 7: “hope”) to create the contig database for the following metagenomics workflow [41].

Genome reconstruction was performed using Semibin (version 1.1.1), a machine-learning-guided software package that combines a semi-supervised approach with deep Siamese neural networks by using an advanced co-assembly binning workflow with a semi-supervised mode [35]. For dereplication and quality filtration of metagenome-assembled genomes (MAGs), we used dRep (version 2.2.3) and CheckM2 (version 1.0.1) with the following parameters: dereplicate: comp 10, con 5, S_algorithm fastANI, sa 0.95, and predict function in case of CheckM2 [42, 43]. It is worth noting that dRep uses CheckM1, so the contamination of nrMAGs may differ from the dRep filtering settings (Supplimentary Table 3). MarkerMAG was used in default mode to detect, assemble, and link 16 S rRNA genes to MAGs and calculate the corresponding copy number (matam_16s: pct 5,10,25,50,75,100 –i 0.99, and link function on default parameters) [44].

Open reading frames (ORFs) were identified by Prodigal (version 2.6.3). InterProScan version 5.31–70 was used to functionally annotate gene coding sequences using the Pfam database [45]. Functional profiles were supplemented using data from the Kyoto Encyclopaedia of Genes and Genomes (KEGG) function modules by Anvi’o (anvi-run-kegg-kofams) [46]. The enzymes involved in carbohydrate utilisation were identified using a combination of Pfam functional profiles and data from the carbohydrate-active enzyme database (CAZy) [47]. We then used the Genome Taxonomic Database (GTDB: Release 207) with GTDB-Tk (version 2.1.1) for taxonomic assignment [48]. Reconstructed non-redundant MAGs (nrMAGs) that showed greater than 90% completeness were also compared with entries in the Biogas Microbiome database [29] using fastANI (version 1.33) [49]. nrMAG statistics are summarised in Supplimentary Table 3.

The abundance values of nrMAGs’ in each sample were calculated with MetaWRAP (version: 1.3.2) quant_bin module (using Salmon), similar to the TPM (transcript per million) calculation process, which here refers to “copies per million reads” (CPM; Supplimentary Table 4) [50]. Reads are aligned to contigs of nrMAGs, and the resulted coverage values were standardised by sample size (for every 1 million metagenomic reads) and by contig length (in nucleotides). Phylogenomic trees were generated using a set of 120 bacterial and 53 archaeal single-copy core genes (SCGs) via GTDB-Tk (version 2.1.1; classify_wf) and IQTree2 (version 2.2.0.3) using the following parameters: number of bootstraps: 1000; maximum iteration: 1000; stopping rule: 100 [51, 52]. The interactive Tree of Life (iTOL: version 6.7.3; https://itol.embl.de/) tool was employed to visualise the phylogenomic tree as well as some binning results.

### Metatranscriptome mapping and analysis

We first extracted ORFs from MAGs using bedtools: getfasta (version: 2.27.1) to obtain MAG-specific gene calls. We then combined these to create a Salmon index (using the parameter keepDuplicates; https://github.com/COMBINE-lab/salmon). Read counts were calculated with Salmon (version 1.8.0) in quasi-mapping mode with GC bias correction (gcBias). The main output file (quants.sf) contains the quantified number of reads (numReads) and its quantity in TPM values (Supplimentary Table 5). The TPM calculation process (used as an activity metric in the present study) was done similarly as mentioned before, taking the effective length-weighted gene (GC bias) and mapped transcriptomic reads per sample corrections.

### Statistical analysis

BMP test results were visualised by *ggplot2* (version 4.1.3), and significant differences between maximum biogas potentials (i.e., from batch tests using α-cellulose) were calculated using the *ggsignif* (version 0.6.4) package (i.e., using a between-pairs Wilcoxon-test, with one-way ANOVAs used to compare multiple groups) [53]. Multidimensional scaling of samples (i.e., MDS plots to display Bray-Curtis dissimilarities), MAG abundance, and activity values were visualised by *microViz* (version 0.9.0) [54]. Eucledian distances and differences among samples were calculated (using permutational multivariate analysis of variance; PERMANOVA: n_perms: 1000) and visualised by the *microeco* R package (version 0.14.1) [55]. To assess the significance of differences between MAGs, we used *lefser* (the R package for linear discriminant analysis effect size calculator; version 4.3) inside *microeco*, with a significance threshold of *p* ≤ 0.05 (trans_diff: alpha = 0.05, p_adjust_method = fdr, lefse_min_subsam = 10, lefse_norm = 1e + 06, boots = 30) [56].

We then analysed the occurrence data to identify the core microbiome. A taxon observed in 83.3% of samples (i.e., in 10 out of 12) with an abundance greater than 1 (~10x genome coverage) was considered to be a member of the core microbiome [37]. The *ggvenn* and *ggtern* packages (versions 0.1.10 and 3.4.1) were used to visualise the core microbiome’s (at species and phylum level) distribution between the BPs [53]. Co-occurrence analysis and correlations between AD parameters and taxonomic IDs of core microbiome members (at the species level or otherwise at the highest taxonomic level) were calculated by *microeco* [55]. *NetComi* (network construction and comparison for microbiome data; version 1.1.0) was utilised for calculating and normalising the association matrix (trans_network: cor_method = pearson, use_NetCoMi_pearson_spearman = TRUE, filter_thres = 0.001, then cal_network: COR_p_thres = 0.01, COR_cut = 0.7) [57, 58]. The *ggraph* package (version 2.1.0) was used to visualise the correlations. To reveal correlations between microorganisms and chemical parameters, the trans_env function was employed (use_data = species, cor_method = pearson, p_adjust_method: fdr).

The results of the metagenomics and metatranscriptomics analyses of carbohydrate-active enzymes were visualised by Circos online (http://circos.ca/) and *ggplot2*. Differential gene expression and enrichment analysis were done by the *DESeq2* package (1.34.0) implemented in R [59]. Salmon quant files (quants.sf) were imported in R with *tximport* (version 1.22.0; settings: type = salmon, txOut = TRUE) (https://github.com/mikelove/tximport). We filtered the counts to retain genes with a minimum of 5 reads across 4 samples obtained from Salmon alignments (numReads) and normalised by *DESeq2* (with default parameters; Supplimentary Table 5). Finally, to visualise significantly different genes, we used the *ggplot2* package (using the following settings: log_2_ FC: ≥2.0, *p* ≤ 0.05).

## Results and discussion

### Different substrates but similar methane yields

Samples were collected from the anaerobic digesters of three industrial-scale BPs (“AD samples” and Supplimentary Table 1). KBP was mainly fed with chicken manure and pre-treated wheat straw; SZBP was fed with pig slurry and maize silage; and MWBP treated municipal wastewater sludge containing diverse materials. All digesters were operated at mesophilic (i.e., 36 °C to 38 °C) temperatures in continuously stirred tank reactors. During the seasonal monitoring period (i.e., four sampling points per BP), all reactors operated stably, and no failures were reported.

**Fig. 1:**
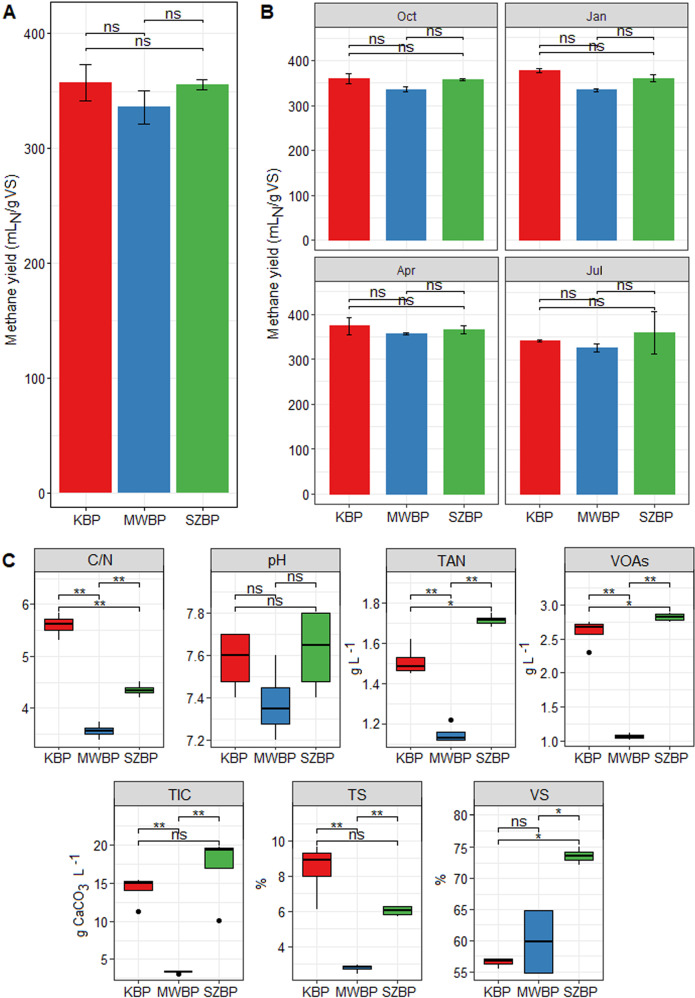
Parameter measurements of the AD chemical process. Different colours represent the three BPs. The Kecskemét biogas plant (KBP) is shown in red, the biogas plant digesting municipal wastewater sludge (MWBP) is shown in blue, and the Szeged biogas plant (SZBP) is shown in green. **A** Results of the standard BMP test measurements of three BPs (see: “AD samples”). **B** BMP methane yields over the tested periods. **C** Anaerobic digestion chemical parameters of the sludge from individual BP digesters (C/N carbon to nitrogen ratio, TAN total ammonia nitrogen, VOAs volatile organic acids, TIC total inorganic carbon, TS total solids, VS volatile solids). Mean differences were analysed via ANOVA and considered statistically significant as follows: *p* < 0.05 (*), *p* < 0.001 (**), ns no significant difference.

Standard BMP tests were performed (see details in “Determination of AD chemical parameters”) to measure the maximum biogas potential of the different AD communities. Methane yields varied from 340 mL (Standard Deviation: 2.6 mL) to 376 mL g VS^−1^ (SD: 14.6 mL). Although the inoculum for BMP tests originated from digesters fed with distinct substrates, the methane yields were highly similar (Fig. 1A). Moreover, tests from the Oct, Jan, Apr, and Jul time points all showed similar methane yields among the three biogas plants (Fig. 1B; for April: KBP = 362 ± 14.6; MWBP = 356 ± 2.4; and SZBP = 365 ± 7.6 mL methane g VS^−1^). In earlier investigations, similar methane potential ranges were observed in BPs digesting diverse types of biomass of agricultural or municipal origin [60–62].

Although the main chemical process parameters were within optimum ranges [63–65], they showed clear differences among the three BP reactors (Supplimentary Table 1). The fallowing parameters were measured: volatile organic acids (VOAs), total inorganic carbon (TIC), total ammonia nitrogen (TAN), carbon-to-nitrogen ratio (C/N), total solids (TS), and volatile solids (VS). Of these, the C/N, VOAs, and TAN concentrations showed the highest variability among the BPs (ANOVA: *p* < 0.05) (Fig. 1C). These values were typically lower in MWBP (*p* < 0.01) compared to those in KBP and SZBP [66].

### High-quality MAGs from the AD microbiomes

Semi-supervised binning complemented with machine learning (ML) is a recently developed approach that has proven to be highly beneficial for extending knowledge of microbial genomics. Earlier research has shown that an ML-based habitat-specific model enhances the metagenome binning process for complex microbiomes, outperforming existing unsupervised nucleotide composition and abundance-based methods (i.e., Metabat2) [35, 36]. However, due to unusual read depth and atypical nucleotide composition, ribosomal RNA genes are frequently absent from MAGs recovered from short-read sequencing data [34, 44]. These genes are essential for studying the genomics and phylogeny of uncultivated microorganisms and permit analyses connecting MAG data with 16 S rRNA gene databases.

**Fig. 2:**
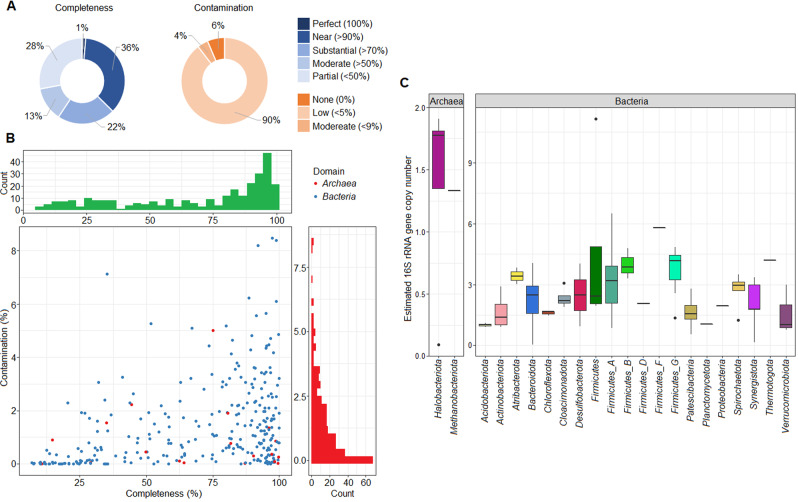
Binning performance and 16 S rRNA gene copy number estimation as deduced from metagenome data. **A** Comparison of the completeness and contamination of the non-redundant metagenome-assembled genomes (nrMAGs) produced by Semibin and analysed by CheckM2 using the default set of SCGs. **B** Distribution of nrMAGs reconstructed by Semibin based on completeness and contamination. Considering dRep employs CheckM1, the contamination of nrMAGs may differ from the dRep filtering settings. **C** Estimated 16 S rRNA gene copy number for 22 phyla (i.e., two *Archaea* and 20 *Bacteria* taxa). For some phyla, it was possible to determine the copy number of one representative: *Methanobacteriota*, *Firmicutes F*, *Firmicutes D*, *Planctomycetota*, *Proteobacteria*, and *Thermotogota*.

In the present study, more than 296 million metagenomic sequence reads passed the filtering step (with an average of 24.6 million reads per sample). Filtered reads were co-assembled by Megahit (three independent assemblies per BP), resulting in a total of 283,491 contigs (KBP: 107,920; MWBP: 98,415; SZBP: 77,156 contigs; for details, see: Suppl. Table 2). A genome-centric metagenomics strategy was followed for each co-assembled contig set. The dereplicated and quality-filtered set of MAGs was then used for further analysis. Semibin produced 297 nrMAGs (nr = non-redundant), of which 107 (36%) had more than 90% completeness. It is noteworthy that 6% of nrMAGs were identified as containing 0% contamination and a further 90% containing >5% contamination by CheckM2 analysis (Fig. 2A and B). Furthermore, 24% of nrMAGs (*n* = 70) were identified at a quality level that was higher than their representatives in the Genome Taxonomy Database (Supplimentary Table 3). These observations confirm the effectiveness of Semibin as a binning procedure for complex anaerobic biogas-producing communities [35]. In addition, the larger number of unique nrMAGs binned by Semibin may also lead to better mapping of metatranscriptome data.

The MarkerMAG program was then used to detect, assemble, and link 16 S rRNA genes from metagenomes to MAGs and to estimate copy numbers (see: Materials and Methods “Metagenome assembly and binning” and Supplimentary Table 3) [44]. In the present study, 16 S rRNA genes (min length: 1200 nucleotides) were detected in 82 nrMAGs distributed across 22 phyla (representing 28% of all nrMAGs). Representatives of the *Firmicutes* possessed the highest estimated mean copy number of this gene (3.6 copies), while members of the *Halobacteriota* and *Methanobacteriota* showed the lowest copy numbers, with an average of 1.3 copies each (Fig. 2C). Along with previous scientific reports, our data suggested that amplicon sequencing of 16 S rRNA genes underestimated the abundance of the archaeal community in AD [67, 68]. One method to better estimate microbial abundance derived from 16 S rRNA gene sequencing is to normalise results via copy number per detected genome [69]. However, the actual 16 S rRNA gene copy number is unknown for many prokaryotes [34]. Current bioinformatic solutions for normalising amplicon sequencing data rely on the apparent phylogenetic conservation of individual gene copies, and this assumption may only be valid for short phylogenetic distances [70]. Genome-resolved metagenomics combined with 16 S rRNA gene detection may help bridge this knowledge gap.

### BPs showed dissimilar microbiome compositions

**Fig. 3:**
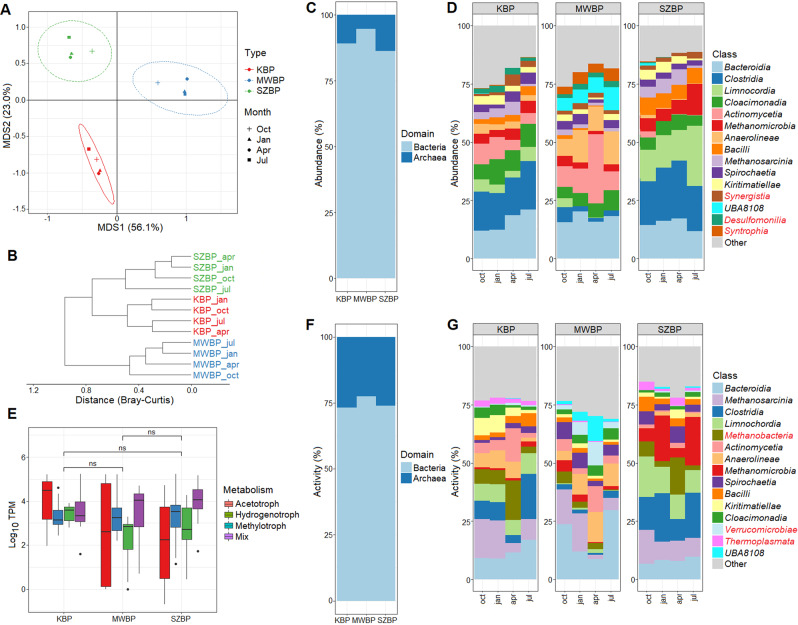
Biogas-producing microbiome abundance (in CPM %) and activity (in TPM %) were analysed using a genome-centric metagenomics and metatranscriptomics framework. **A** Multidimensional scaling of samples. This measure indicates the Bray-Curtis dissimilarities of the microbial community at the level of species annotation or higher. Each symbol is related to a specific BP sample. Oct, Jan, Apr, and Jul, indicate the months in which the samples were collected. **B** Euclidian distance of BP samples. This measure represents the dissimilarity of microbial communities at the level of species annotation or higher. **C** Taxonomic distribution of nrMAGs at the level of domain for the three BPs (in CPM %). **D** Taxonomic distribution of nrMAGs. This shows the most abundant taxa in all twelve samples taken (i.e., each BP-time point pair) resolved to the class taxonomic level (shown in CPM %). The red names indicate that they are not in the top 15 most active classes. **E** The overall metatranscriptomic activity of archaeal microorganisms involved in different methanogenic pathways (mix: indicates those nrMAGs that are capable of using all three methanogenic pathways). **F** Cumulative metatranscriptomic activity of nrMAGs at the domain taxonomic level for the three BPs (shown in TPM %). **G** The metatranscriptomic activity of nrMAGs. Shown are the most active taxa present in all twelve samples taken (i.e., each BP-time point pair) resolved to the class taxonomic level (shown in TPM %). The red names indicate that they are not in the top 15 most abundant classes.

Overall, we found that the three AD reactors harboured distinct microbial communities. Multidimensional scaling (MDS; implemented using the Bray-Curtis dissimilarity measure) revealed that the variance in microbiome composition across the three biogas plants was significantly larger (PERMANOVA: *p* = 0.001) than the variance in microbiome structure across different sampling points of each individual biogas plant. The microbiomes of the different BPs showed characteristic changes over time, but in each case, these changes were distinctive for that plant (Fig. 3A). This finding was confirmed by Euclidean distance calculations, which indicated that the microbiomes of KBP and SZBP were more similar to each other than to MWBP (Fig. 3B). In accordance with past research, our analyses confirmed that the KBP and SZBP microbiomes predominantly contained *Bacteroidia*, *Clostridia*, and *Limnochordia*, whereas the MWBP microbiome contained *Bacteroidia*, *Anaerolineae*, and *Actinomycetia* (Fig. 3D) [29, 31, 71]. We can therefore conclude that there is a common microbial community landscape that includes the main taxa found in the biogas digesters studied here [3, 4].

Examination of microbial abundance and activity using metagenome and metatranscriptome data distributions revealed distinct patterns. Based on relative abundance (CPM %), the top three classes found were *Bacteroidia*, *Clostridia*, and *Limnochordia*, although the activity (TPM%) of *Methanosarcina* outperformed members of these classes. Moreover, we also found striking differences in rank and taxonomic composition among the top 15 most active nrMAGs compared to the most abundant microbial classes (Fig. 3D and G). Thus, the relative abundance of microorganisms is divergent from relative metatranscriptomic activity. Overall, the representatives of the domain *Archaea* showed higher activities than abundance, as evidenced by comparing TPM% to CPM% values (Fig. 3C and F; Supplimentary Tables 4 and 5). This phenomenon was also observed in an anaerobic biogas-producing community [72].

The methanogenic archaea identified through metatranscriptomics exhibited no significant differences in overall activity across all three industrial-scale biogas plants (Fig. 3E). This observation indicates that the methanogenic archaea exhibit comparable functionality, notwithstanding variations in the conditions and parameters across the different biogas facilities. This resilience may be due to the diversity of methanogenic archaea in the digesters, which can provide redundancy to the methane producing community [19, 73–75].

### Biogas-producing communities and the microbial dark matter

**Fig. 4:**
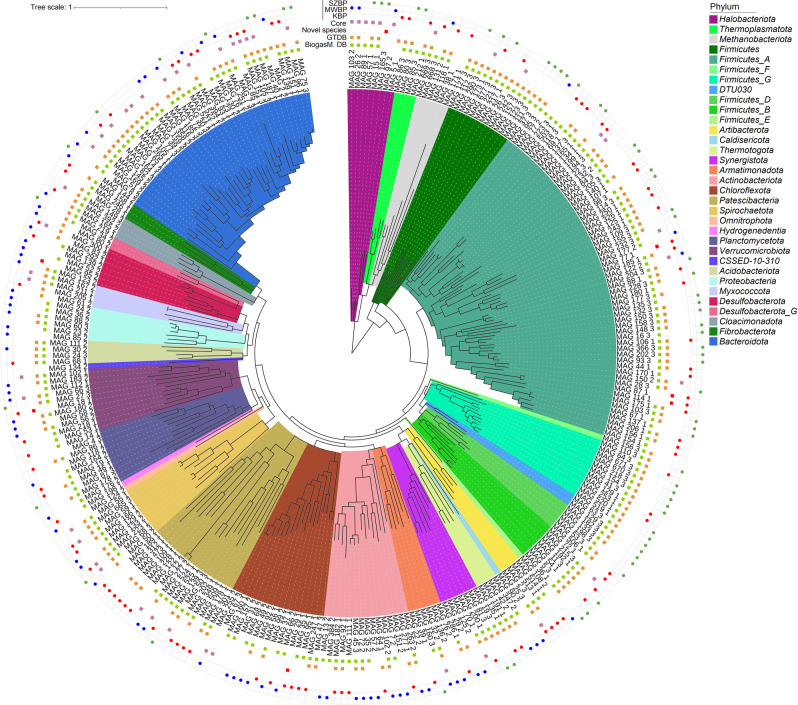
Phylogenetic tree of reconstructed nrMAGs based on bacterial and archaeal SCGs. The background colour of the inner phylogenetic tree marks the phylum that they belong to the first ring shows the nrMAG number. In the next two rings, the symbols represent the presence of specific nrMAGs in the Biogas Microbiome (green) and GTDB (orange) databases. The red symbols mark the seven nrMAGs that have high completeness (>90%), low contamination (<5%) and have not been found in any available databases (*Archaea*: MAG_165_3; *Bacteria*: MAG_114_1, _18_1, 29_1, 77_1, 87_1 and 95_2). Purple symbols represent the core nrMAGs. The outer ring represents bacteria that are significantly more prevalent in the particular BP (using *lefser, p* < 0.05).

Based on known lineage-specific marker gene sets (SCGs) and on MIMAG data, we detected 36% high-, 34% medium-, and 30% low-quality nrMAGs [76]. The Genome Taxonomy Database (GTDB) was then employed for the taxonomic assignment of reconstructed nrMAGs. These results showed that nrMAGs could be categorised into 33 phyla, of which three belonged to the *Archaea* and 30 to the *Bacteria* (Fig. 4 and Supplimentary Table 3).

The nrMAGs were compared against the Biogas Microbiome database, utilising the fastANI tool to calculate average nucleotide identity (ANI) [29]. This database contains a comprehensive set of microbial genomes previously found in biogas digesters. We found that 83% of the reconstructed high-quality nrMAGs (completeness >90%; *n* = 107) were present in the Biogas Microbiome database (ANI cutoff: 95%). It is worth noting that seven high-quality nrMAGs, which accounted for 7% of the total high-quality nrMAGs (*n* = 107) and 2% of all nrMAGs (*n* = 297), could not be associated with a nearest representative in either database. These nrMAGs were deemed novel because they did not meet the following criteria: species-level identification with 95% reference radius for GTDB and ≥95% ANI for Biogas Microbiome (Fig. 4 and Suppl. Table 3). Three high-quality nrMAGs were found to contain 16 S rRNA gene sequences as well (Supplimentary Table 3). In addition, the seven putative novel nrMAGs demonstrated high abundance and activity, accounting for 3% of the total count per million (CPM) and 5% of the total transcripts per million (TPM), respectively, in the examined microbiome. These nrMAGs may be members of the hypothesised microbial dark matter that can now be released into the realm of known participants in AD communities.

### AD chemical parameters and the core community

**Fig. 5:**
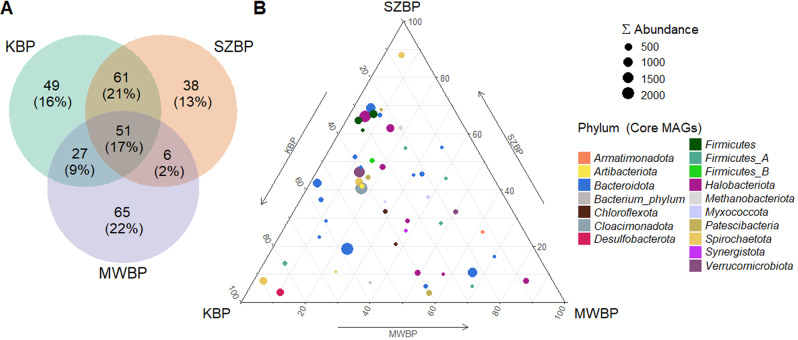
Analysis of core microbiome distribution. **A** Venn diagram indicates the total number of nrMAGs and the number of shared nrMAGs among the three BPs (percentages as indicated). **B** Ternary plot showing the distribution of bacterial and archaeal nrMAGs between BPs. The dot colour represents the phylum that the nrMAGs belong to. The size of the dot is proportional to the total abundance (i.e., cumulative CPM value in all BPs) of specific nrMAGs.

Genome-centric metagenomics-based core microbiome analysis was used to identify potentially relevant taxa that may play a role in shaping the core microbial community. This macroecological study was therefore supported by metatranscriptome data (Fig. 5 and Supplimentary Fig. 1) [37]. These analyses provide insight into the relationship between nrMAGs and AD parameters, as well as their roles in shaping the core microbiome.

The examination of the distribution of reconstructed nrMAGs in specific biogas plants revealed that MWBP possessed the most unique microbiome of the three investigated systems. KBP and SZBP showed somewhat overlapping microbiomes, as evidenced by the MDS and Euclidean distance calculations. Nonetheless, we detected 51 nrMAGs in all three digesters (Fig. 5A). Most of the core nrMAGs identified were representatives of well-known hydrolysing bacteria (e.g., *Firmicutes*, *Bacteriodota*) and methanogens with versatile metabolic activities (e.g., *Halobacteriota*, *Methanobacteriota*). These microorganisms play an important role in maintaining biogas productivity and sustainable system performance [14, 77]. Although some nrMAGs were present in all digesters, their abundance varied considerably. Among core bacteria, the phyla *Firmicutes, Firmicutes A*, and *Bacteriodota* were found in both SZBP and KBP, while members of the *Verrucomicrobiota*, *Armatimonadota*, and *Chloroflexota* predominated in MWBP [6, 29, 66] (Figs. 4 and 5B).

**Fig. 6:**
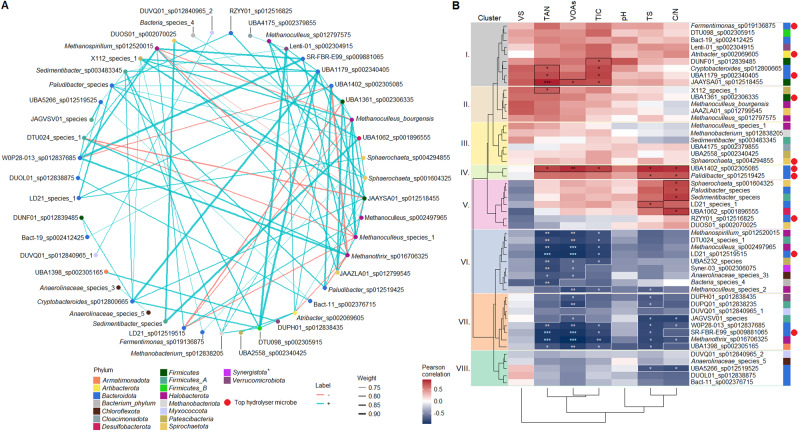
Correlation analysis of core nrMAGs. **A** Pearson correlations between core nrMAGs. The robustness is calculated under the adjusted significant *p* value is ≤0.05 and the correlation index (Pearson’s rho) is > 0.7 based on network. The blue lines represent positive correlations (Pearson’s rho > 0.7), red lines represent negative correlations (Pearson’s rho < −0.7). A phylum marked with an asterisk is not present among the correlating microorganisms. **B** Pearson correlations between core nrMAGs and measured AD chemical parameters. Asterisks represent the significant correlations (considered statistically significant as follows: *p* < 0.05 (*), *p* < 0.001 (**), *p* < 0.0001 (***), ns (no significant difference)). Using the cladogram shown on the left and data for TAN (total ammonia nitrogen), VOAs (volatile organic acids), TIC (total inorganic carbon), TS (total solids) and C/N (carbon to nitrogen ratio), eight clusters were distinguished. These are shown here separated by colour as follows: Cluster I: grey; Cluster II: brown; Cluster III: yellow; Cluster IV: green; Cluster V: purple; Cluster VI: blue; Cluster VII: orange; and Cluster VIII: dark green. Coloured boxes represent the specific phyla to which the nrMAGs belong to. Red dots represent the top hydrolysing nrMAGs (*n* = 10).

A co-occurrence analysis of nrMAGs was performed and their correlations with AD chemical parameters were calculated (“Statistical analysis”) (Fig. 6). The co-occurrence network analysis demonstrated that the majority of methanogens exhibited a positive correlation with the phyla *Bacteroidota* and *Firmicutes*. The main parameters influencing the abundance of core microorganisms were TAN, VOAs, and TIC (Pearson’s rho > 0.5). Based on this observation, eight clusters showing characteristic correlations with AD chemical parameters were plotted (Fig. 6B). The eight clusters can be divided into two groups, with microorganisms from clusters I–V correlating positively with the main influencing parameters and microorganisms from clusters VI–VIII correlating negatively with these parameters. We also found that top core nrMAGs belonging to the phyla *Firmicutes*, *Spirochaetota*, and *Methanobacteriota* were positively correlated with TAN, VOAs, and TIC, while members of the phylum *Bacteroidota* were also correlated with many of the measured parameters. Finally, the C/N ratio showed a more pronounced impact on *Bacteroidota* than *Firmicutes* among the top core nrMAGs (Fig. 6B).

The *Methanoculleus* genus (phylum *Halobacteriota*, representing hydrogenotroph methanogens) was found to be a diverse community in the core microbiome. The representative species of *Methanoculleus* that exhibited a positive correlation with TAN and VOA concentrations were predominant in KBP and SZBP, whereas those with a negative correlation were more prevalent in MWBP. Additionally, they co-occurred with members of the phyla *Bacteroidota* and *Firmicutes*. Microorganisms belonging to the phyla *Bacteroidota* and *Firmicutes* can produce organic acids, alcohols, hydrogen (H_2_), and carbon dioxide (CO_2_) via acidogenesis and acetogenesis, and many of these microorganisms exist in syntrophy with acetotrophic and hydrogenotrophic methanogens. The hydrogenotrophic methanogens consume hydrogen via interspecies hydrogen transfer (IHT) and use the energy gained from the reduction of CO_2_ to methane [78]. The hydrogen-consuming methanogenic microorganisms can rapidly scavenge hydrogen and maintain the partial pressure of hydrogen at a low level. This leads to a thermodynamically favourable condition for the hydrogen-producing acetogenic bacteria to break down organic compounds into acetate, H_2_, and CO_2_ [14, 79, 80].

Two species of methanogenic archaea, *Methanoculleus* sp002497965 and *Methanospirillum* sp0125200015 (designated MAG_26_2 and MAG_103_2), were found in Cluster VI and showed a negative correlation with *Methanoculleus bourgensis* and *Methanoculleu*s species 1 belonging to Cluster II and III. MAG_26_2 and MAG_103_2 were commonly present in the MWBP microbiome (Fig. 4). *Methanoculleus* sp002497965 and *Methanospirillum* sp0125200015 were originally identified in AD microbiomes, but to the best of our knowledge, detailed information about these microorganisms remains unavailable [81–84].

In the present study, MAG_26_2 and MAG_103_2 were classified as strict hydrogenotrophic methanogens by metatranscriptomics data. Moreover, they exhibited a negative correlation with TAN and VOA concentrations (Pearson’s rho > −0.7). Based on genomic data, these archaea belong to the class of methanogens capable of maintaining hydrogenotrophic methanogenesis using formate as an electron donor. Typically, formate is oxidised to CO_2_ by formate dehydrogenase, upon which it is further reduced to methane [85]. Here, interspecies formate transfer (IFT) was maintained by microbial partners, namely SR-FBR-E99 sp002497965 and LD21 sp012519515 (from the phylum *Bacteroidota*; MAG_72_3 and MAG_394_2), which were positively correlated with MAG_26_2 and MAG_103_2 (Fig. 6A). These MAGs have been previously detected in biogas digesters and were described for their versatile hydrolysing ability. Aside from carbohydrate utilisation, they are protein- and amino-acid degrading microorganisms capable of producing formate. They were also consistently detected alongside formate-utilising methanogens [86].

Among the abundant core nrMAGs present, we identified *Methanothrix* sp016706325 (MAG_97_2), an archaeon that is apparently capable of acetotrophic methanogenesis (Supplimentary Fig. 1). Based on its MAG metatranscriptome profile, it primarily performs acetotrophic methanogenesis and can take up electrons via direct interspecies electron transfer (DIET) to drive CO_2_-reducing methanogenesis. Our data indicate that it possesses highly active acetyl-CoA and F_420_ biosynthesis pathways as well as active, membrane-associated electron transfer (cytochrome C transmembrane protein; Supplimentary Table 4). According to a previous study, *Methanothrix* species exhibit greater activity when deriving a portion of their energy from DIET as opposed to relying solely on acetate. However, information about their natural syntrophic partners is limited [87]. This archaeon (with *Methanospirillum* sp0125200015), is closely associated with *Methanoculleus* sp002497965. This archaeal network has been detected to co-occur with the previously mentioned amino-acid degrading microorganisms (Fig. 6A). These microorganisms also have active C-type cytochromes and pili synthesis ability, both of which are essential for DIET (MAG_72_3 and MAG_394_2; Supplimentary Table. 5) [79, 88–90]. During metabolic processes, electrons can be generated and carried by reducing equivalents such as reduced ferredoxin. To re-oxidise these electron carriers, formate production may allow the use of electron disposal routes to conserve energy [86]. Thus, *Methanothrix*_sp016706325 (MAG_97_2) may play an essential role in the biogas-producing community by supporting electron disposal routes [91]. Based on data from the scientific literature, as well as a comprehensive analysis of anaerobic digesters in Danish wastewater treatment plants, representatives of *Methanothrix* showed a negative correlation with both acetate and TAN concentrations [66, 92, 93].

Correlations between microbial communities and chemical process parameters imply a distinct electron transfer mechanism during the AD of agricultural biomass and wastewater. These processes are negatively correlated with VOAs and TAN levels and commonly occur when electron disposal routes are abundant. For example, our data and previous studies suggest that protein hydrolysing and amino-acid-degrading microorganisms – which according to the GTDB taxonomy belong to the phylum *Bacteroidota* and family VadinHA17 (Supplimentary Table 3) – build syntrophic relationships with hydrogenotrophic methanogens to maintain formate production and dispose of electrons via DIET [86].

### Top hydrolysers

**Fig. 7:**
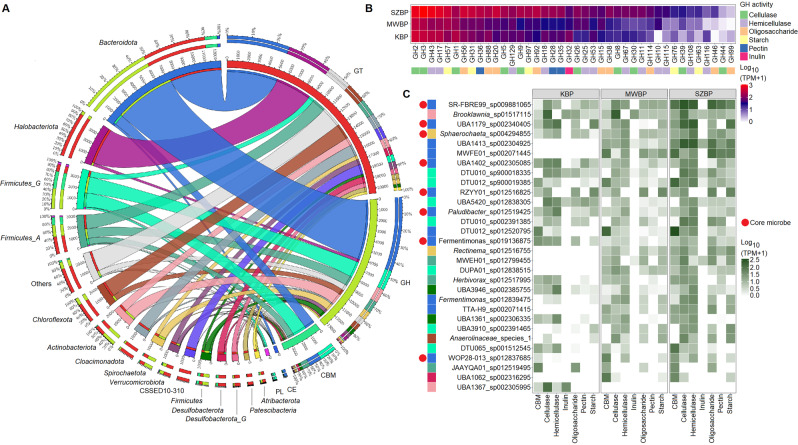
CAZymes identified in metatranscriptomic data and associated taxa. **A** Circos plot illustrating the identified CAZyme classes and their activity distribution across bacterial phyla. The enzymes were grouped into five CAZyme classes. Glycosyltransferases (GTs) showed the highest activity, followed by glycoside hydrolases (GHs), carbohydrate-binding modules (CBMs), carbohydrate esterases (CE), and polysaccharide lyases (PL). Overall, GH and GT activity were detected in all observed microbial phyla. Most (51% TPM) CBM activity was linked to the *Firmicutes* and *Bacteroidota*. **B** Heatmap of widely distributed and common glycoside hydrolase (GH) enzyme families. GH activity is specified by coloured boxes. **C** Heatmap of the activities of the carbohydrate-binding module (CBM) and glycoside hydrolase (GH) enzyme families for the top 30 microbial species (shown in log_10_ TPM values). Red dots indicate specific nrMAGs present in the core community (*n* = 10).

Carbohydrate-active enzymes (CAZymes) are responsible for the decomposition of polymeric carbohydrates. To characterise this rate-limiting step, the metatranscriptome dataset was queried for various CAZymes and signs of CAZyme activity using a combined dataset based on the Pfam and CAZy databases (Fig. 7). Identified CAZymes were then linked to nrMAGs (see: “Metagenome assembly and binning”).

Among the CAZymes identified, the glycoside hydrolases (GHs) are a major enzyme family involved in the degradation of complex carbohydrates (Fig. 7A) [94]. This diverse enzyme family includes cellulases, hemicellulases, pectin, inulin, and various oligosaccharide- and starch-degrading enzymes. According to the metatranscriptome data, cellulase, hemicellulose, and starch-degrading enzymes showed elevated activity in KBP and SZBP (Fig. 7B, C). Among several differences, the activities of cellulases (GH2 and GH3), hemicellulases (GH43 and GH18), and starch degrading enzymes (GH97 and GH31) were all significantly higher in KBP and SZBP compared to those in MWBP (log_2_ FC: ≥2; *p* ≤ 0.05) (Fig. 7B and Supplimentary Table 5). Taxonomically, members of the phyla *Bacteroidota* (representing 46% TPM of GHs), *Firmicutes G* and *Firmicutes A* (12% and 7% TPM of GHs, respectively) were the dominant contributors of GH activity. These phyla have been found to be the main polysaccharide degraders in many biogas-producing communities [77, 95]. Nevertheless, our metatranscriptomics data suggested that members of other phyla such as *Actinobacteriota* (8% TPM of GHs), *Chloroflexota* (4% TPM of GHs), and *Spirochaetota* (5% TPM of GHs) also expressed complex carbohydrate-degrading enzymes (Fig. 7A) [29, 38]. A combined quantitative assessment of carbohydrate-binding modules (CBM) and GH activity revealed that the top 30 hydrolysing nrMAGs are distributed across multiple phyla (Fig. 7C).

Eight core nrMAGs were detected among highly active hydrolysing nrMAGs. These eight represented about 35% of the total top hydrolysers activity (Fig. 7C). We found that UBA1179_sp002340405 (MAG_187_1) and *Fermentimonas* sp019136875 (MAG_96_1) belong to Cluster I, UBA1402 sp002305085 (MAG_105_3) and *Paludibacter* sp012519425 (MAG_151_3) to Cluster IV, and SR-FBR-E99 sp009881065 (MAG_72_3) and W0P28-013 sp012837685 (MAG_251_2) to Cluster VII. (Figs. 6B and 7C). Members of the top core hydrolysers belonging to Cluster IV and I showed the most widespread interactions (Fig. 6A). These clusters were directly or indirectly involved via IHT and showed positive associations with *Methanoculleus bourgensis*, *Methanoculleus* species 1, *Methanobacterium* sp012838205, and *Methanoculleus* sp012797575 (Pearson’s rho 0.6–0.9). However, the cluster also showed a negative correlation with *Methanospirillum* sp0125200015, *Methanothrix* sp016706325, and *Methanoculleus sp002497965*. The latter hydrogenotroph and acetotroph methanogens capable of IFT and DIET co-occurred with Cluster VII (Pearson’s rho < −0.6). In general, concentrations of TAN, VOAs, and TIC positively correlated with the most active core hydrolysing nrMAGs in Clusters I and IV (Pearson’s rho > 0.6). However, there were exceptions involving microorganisms belonging to Cluster VII.

### Methanogenesis is the most active pathway in the transcriptome

**Fig. 8:**
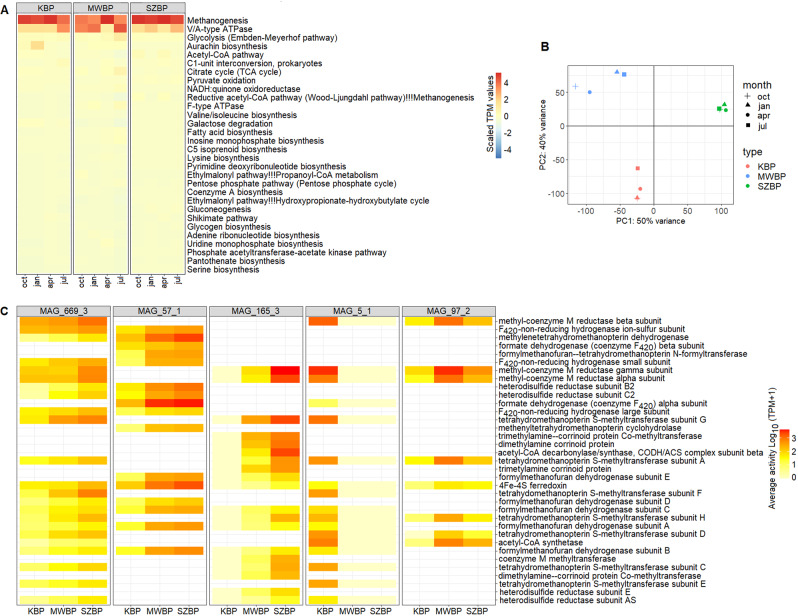
Functional analysis of the three BPs. **A** Heatmap of the most active KEGG modules. Shown are the top 30 KEGG modules from all twelve samples based on metatranscriptomic data (shown in TPM). **B** Principal component analysis of KEGG module functional data. Each symbol is related to a specific BP-sample pair. **C** Significantly different methanogenic genes identified in the five most active methanogens. Shown are differences considered to be statistically significant as calculated by *DESeq2* (i.e., with log_2_ FC > 2.0, *p* < 0.05). The heat map depicts the average gene activity of each methanogen in the various BPs. The blanks represent genes that were not significantly different in the provided methanogen, or the indicated gene is absent in the given nrMAG.

To further characterise the methane-producing food chain, we performed functional analysis of the nrMAGs by combining metatranscriptome data and data from the KEGG database (Fig. 8). The analysis of KEGG modules indicated that the methanogenesis pathway was the most active among all main pathways (mean = 35% TPM of all KEGG pathways). Previous metagenome studies have considered methanogenesis to be a “rare module” in the biogas production community [18, 29, 38] (Fig. 8A). It is important to note that while methanogenesis was indeed a rare module based on the metagenome data, the transcriptome data suggested the opposite [95–98]. Considering this, metatranscriptome analyses, which quantify the biological activities of microorganisms in complex environments, provide a more accurate representation of microbial life and microbial activity occurring within the community than metagenomics studies.

We identified pyruvate-oxidation, the Embden-Meyerhof pathway, and the pentose-phosphate pathway as central carbohydrate metabolism modules. These pathways have been referred to as “core modules” in previous metagenomic investigations [29, 38]. Sugars, coming from hydrolysis, can be converted to pyruvate via the Embden-Meyerhof and pentose-phosphate pathways to produce CO_2_ and electrons. In addition, the acetyl-CoA pathway, fatty acid biosynthesis, and beta-oxidation are active among the top 30 pathways related to carbon fixation, as has been previously observed in manure-supplemented reactors [29]. This finding was supported by the identification of numerous active modules associated with energy, amino acid, and cofactor production, all of which are essential for a well-functioning biogas generation ecosystem. Next, we discovered that KBP and SZBP shared a similar microbial composition and functional profile, while those of MWBP appeared (based on KEGG modules) to be clearly distinct (Fig. 8B).

To assess transcript-level expression patterns, differential expression analysis was used (see: “Statistical analysis”). We detected alterations in 11,383 genes among the three BPs using this high-resolution analysis (log_2_ FC > 2.0, *p* ≤ 0.05) (Supplimentary Table 5). KBP and SZBP were shown to contain fewer differentially expressed genes (DEGs) than these compared to MWBP (Fig. 8B and Supplimentary Fig. 2). A comprehensive investigation of DEGs found 215 genes involved in methanogenesis. The activities of alpha, beta, and gamma subunits of methyl coenzyme M reductase showed the most substantial changes (log_2_ FC > 10, *p* < 0.0001). Additionally, the expression of genes implicated in hydrogenotrophic methanogenesis, such as the ion-sulphur subunit of F_420_-non-reducing hydrogenase and methylenetetrahydromethanopterin dehydrogenase, displayed substantial differences (log_2_ FC > 5, *p* < 0.001). Acetyl-CoA synthetase demonstrated variances in expression across genes involved in acetotrophic methanogenesis (log_2_ FC > 5, *p* < 0.001) [99]. The majority of the different gene activities in KBP were related to *Methanothrix A harundinaceae D* (MAG_5_1), while *Methanothrix*_sp016706325 (MAG_97_2) in MWBP and *Methanobacterium* sp012838205, *Methanoculleus* sp12797575, and *Methanosarcina* species (MAG_669_3, _57_1, and _165_3) in SZBP (Fig. 8C).

In a complex biogas-producing community, methanogenic archaea utilise various metabolic pathways and gene sets to produce methane as a way of obtaining energy and compensating for their abundance through their activity. According to earlier research, under hydrogen-limited conditions, *Methanococcus maripaludis* displayed elevated mRNA levels for genes encoding enzymes including F_420_-non-reducing hydrogenase and methylenetetrahydromethanopterin dehydrogenase, which resulted in an increase in its growth rate [100]. Similar results were observed in our study with the abundant *Methanoculleus* sp12797575 (MAG_57_1) in SZBP. These enzymes demonstrated elevated activity in this hydrogenotrophic methanogen belonging to Cluster II, which co-occurred with JAAZLA01 sp012799545 and UBA1361 sp002306335 hydrolyser nrMAGs (Fig. 6A). The elevated expression of formate dehydrogenase indicates that MAG_57_1 utilises formate as an alternative hydrogen source (Fig. 8C) to compensate for its methanogenic activity. This activity is comparable to the mixotrophic *Methanosarcina* species commonly found in SZBP (which are capable of conducting all methanogenic pathways, however, no species-level representative was identified in the GTDB and Biogas Microbiome databases; MAG_165_3). This is in line with the findings of previous observations that the metabolic diversity of the methanogenic community is critical for efficient biogas production [74, 75].

## Conclusions

In this study, we reconstructed high-quality nrMAGs (non-redundant metagenome-assembled genomes) and conducted a precise metatranscriptome analysis with the assistance of an artificial neural network binning workflow on AD samples taken from industrial-scale biogas reactors. Although the three industrial-scale BPs operated with well-characterised and distinct feedstocks, we observed no differences in their respective methane yields over the observation period. The microbiome composition and the functional repertoires of the KBP and SZBP BPs were more similar to each other than either was to that of MWBP. Multiple bacterial phyla were identified as major hydrolysing microorganisms. Significant correlation between the core microbiome and fermentation parameters such as TAN, VOAs, and TIC suggests that the interaction network in the AD core microbial community is influenced by various chemical operational parameters. Hydrogenotrophic methanogens (*Archaea*: *Halobacteriota*, *Methanobacteriota*) were dominant and positively correlated with the presence of representatives of the bacterial phyla *Firmicutes* and *Bacteroidota*, with whom they engage in versatile interspecies transfers. Distinct electron transfer mechanisms used by hydrogenotrophic methanogens in AD have also been found in agricultural biomass and wastewater. A key archaeal species (*Methanothrix* sp016706325; MAG_97_2) was detected in the core methanogenic community and likely plays a key role in shaping the methane-producing microbial strategy. Our study found that the methanogenic archaea in three industrial-scale biogas plants maintained similar overall activity despite differences in operating conditions and parameters measured. The presence of a diverse range of methanogenic archaea in digesters may enhance community resilience by offering redundancy and functional stability, emphasising the crucial role of metabolic diversity in ensuring efficient biogas production. This finding was also consistent with our BMP test measurements. However, the present case study confirms that important knowledge gaps remain in our understanding of the activities and interspecies relationships between members of the biogas-producing communities. These gaps can be addressed, at least in part, by a framework that combines genome-resolved metagenome analysis with a parallel metatranscriptomics approach that is guided by specific machine-learning algorithms, such as habitat-specific models.

## Supplementary information

Supplementary material

Supplemenatry figure 1

Supplementary figure 2

Supplementary table 1

Supplementary table 2

Supplemenatry table 3

Supplementary table 4

Supplementary table 5

## Data Availability

The raw metagenome and metatranscriptome sequences generated and analysed during the current study were deposited in the NCBI SRA under accession number PRJNA929705. High-quality (compl. >90% cont. <5%) nrMAGs are deposited in the NCBI SRA under accession numbers from SAMN32989584 to SAMN32989690. The main data generated or analysed for this study are included in this published article and its supplementary information files. Workflows and R scripts are available from the first author on reasonable request.
