# Supplementary material for: Inter-kingdom interactions and stability of methanogens revealed by machine-learning guided multi-omics analysis of industrial-scale biogas plants
Source: ISME J. 2023 Jun 7;17(8):1326–39. doi: 10.1038/s41396-023-01448-3 (PMC10356833; doi:10.1038/s41396-023-01448-3)
Supplement: Supplementary file 1 — Supplementary material [file 41396_2023_1448_MOESM1_ESM.docx]

**Supplementary material description for:**

**Inter-kingdom interactions and stability of methanogens revealed by machine-learning guided multi-omics analysis of industrial-scale biogas plants**

**Supplementary Table 1:** Summary data of biogas plants. Shown are all main parameters for the studied biogas plants.

**Supplementary Table 2:** Sequencing statistics. Shown are raw and filtered DNA and RNA summary data. RIN values for RNA are also given in the table. Short DNA sequences were assembled by Megahit (see section 2.5). Assembly statistics created by Quast are also shown.

**Supplementary Table 3:** nrMAG statistics. This table shows the main parameters of the reconstructed nrMAGs, including completeness, contamination, genome size and N50. Contamination and completeness of nrMAGs was estimated both by CheckM1 and CheckM2. All nrMAGs were taxonomically analysed using the GTDB database (see section 2.5) and the results are summarised in this table. Percentages of ANI, MAG code and name in Biogas Microbiome are given. Also shown is a novelty analysis based on the GTDB and Biogas Microbiome databases, which includes only those nrMAGs that show both greater than 90% completeness and less than 5% contamination (based on SCGs). MarkerMAG was used to estimate the 16S rRNA gene copy number of MAGs. The final column describes the MAG data improvement compared to GTDB (release 207).

**Supplementary Table 4:** Relative abundances of nrMAGs. Shown are summary data of the relative abundances of nrMAGs expressed in genome copies per million mapped reads (CPM) values.

**Supplementary Table 5:** Activity of nrMAGs. The MAG taxonomy table contains identified genes found in nrMAGs based on data from the Pfam and KEGG databases. Also shown are the activities of these genes as expressed in read count (columns 12–23) and TPM (columns 24–35). NA refers to hypothetical proteins predicted by Prodigal. The coordinates of the identified genes on the contigs are listed in columns 1–3. Column 11 contains the nrMAG number that the indicated gene belongs to.

**Supplementary Figure 1:** Heat map of the core nrMAGs metatranscriptome. Shown are summary data for the core nrMAGs metatranscriptomics dataset. KEGG modules are listed in order of descending activity. The core microbe names are shown at the bottom of the figure. Empty boxes indicate that a specific activity was not found in a specific nrMAG.

**Supplementary Figure 2:** Volcano plot of the set of genes that are significantly differentially expressed (Log_2_ FC > 2, *p* ≤ 0.05). The figure details the number of DEGs found. The black dots represent genes that are associated with methanogenesis. The red dots represent other genes. The grey dots represent genes that showed no difference between the two BPs tested.
