# Supplementary figures and images for: Inter-kingdom interactions and stability of methanogens revealed by machine-learning guided multi-omics analysis of industrial-scale biogas plants
Source: ISME J. 2023 Jun 7;17(8):1326–39. doi: 10.1038/s41396-023-01448-3 (PMC10356833; doi:10.1038/s41396-023-01448-3)

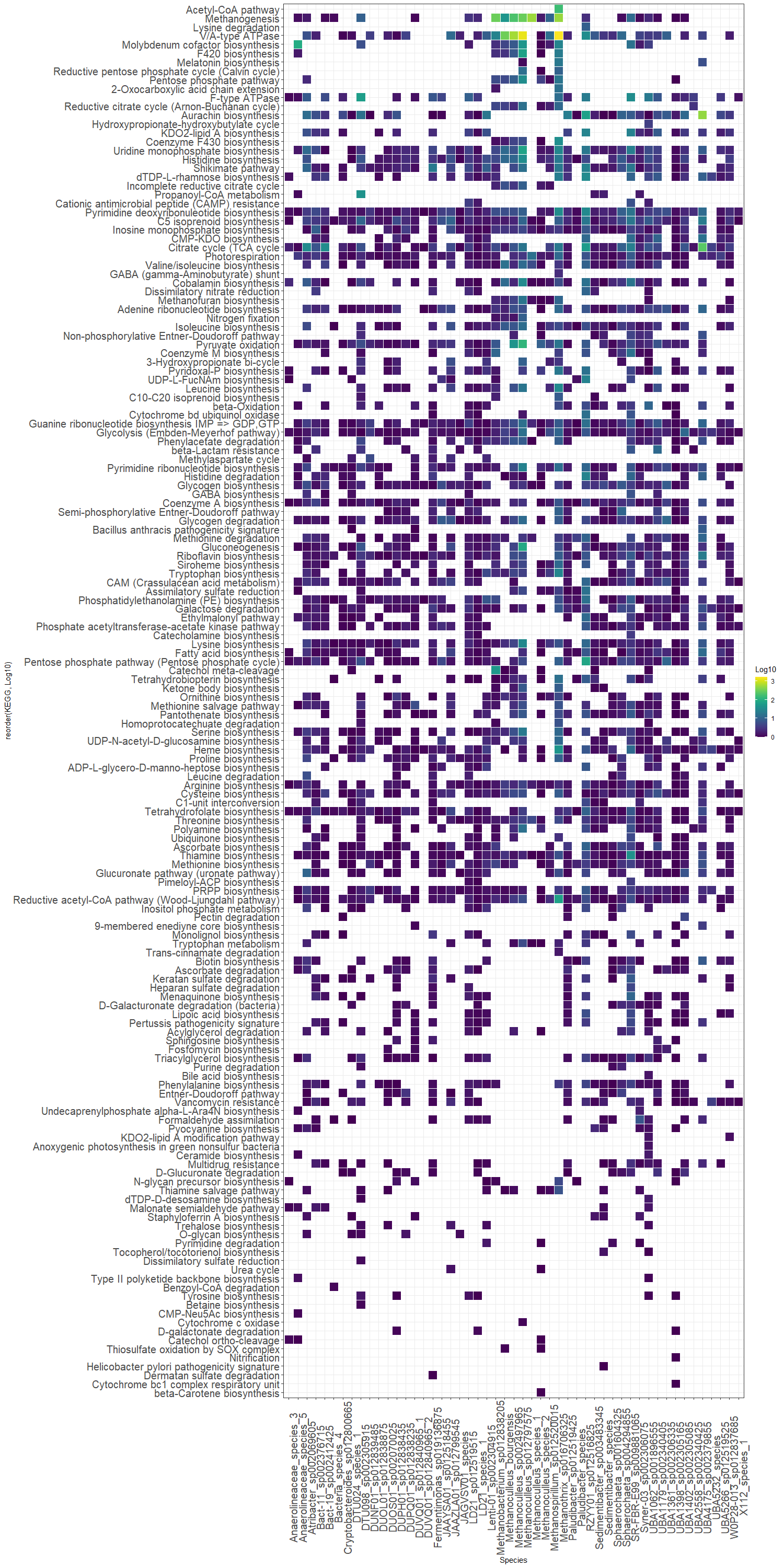

Supplement: Supplementary file 2 — Supplemenatry figure 1 [file 41396_2023_1448_MOESM2_ESM.jpg]

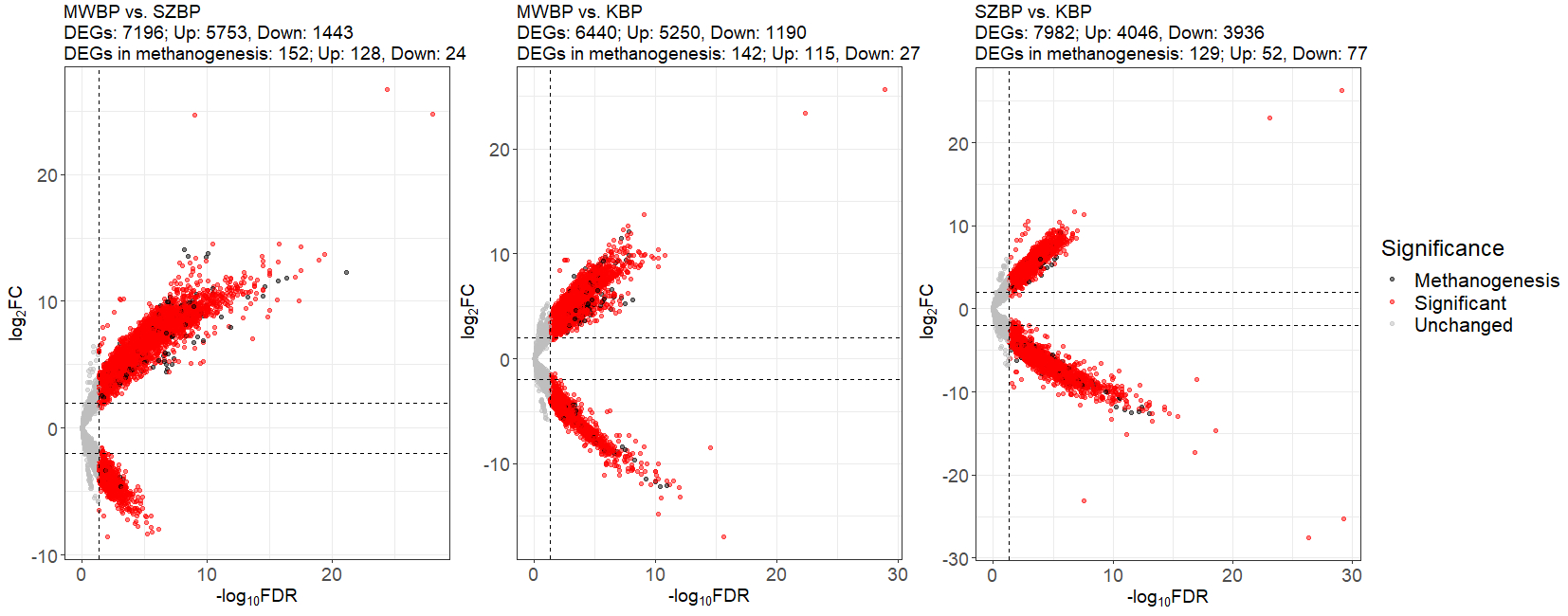

Supplement: Supplementary file 3 — Supplementary figure 2 [file 41396_2023_1448_MOESM3_ESM.jpg]
